# ENO1 functional plasticity linking metabolism with gene regulation and immunity at the host–pathogen interface

**DOI:** 10.3389/fimmu.2026.1979935

**Published:** 2026-10-28

**Authors:** Conghui Zhao, Peilin Li, Wenjun Shi, Shujie Ma

**Affiliations:** 1 Key Laboratory of Animal Pathogen Infection and Immunology of Fujian Province, College of Animal Sciences, Fujian Agriculture and Forestry University, Fuzhou, China; 2 Joint Laboratory of Animal Pathogen Prevention and Control of Fujian-Nepal, College of Animal Sciences, Fujian Agriculture and Forestry University, Fuzhou, Fujian, China; 3 Key Laboratory of Fujian-Taiwan Animal Pathogen Biology, College of Animal Sciences, Fujian Agriculture and Forestry University, Fuzhou, China; 4 Animal Quarantine Laboratory, Technology Center of Qingdao Customs, Qingdao, China

**Keywords:** ENO1, gene regulation, host–pathogen interaction, innate immunity, metabolic reprogramming, multifunctional hub

## Abstract

Enolase 1 (ENO1) is a highly conserved glycolytic enzyme that exhibits remarkable functional plasticity beyond its canonical metabolic role. Increasing evidence indicates that ENO1 participates in diverse cellular and host–pathogen processes, including metabolic adaptation, gene regulation, signaling, immune responses, and pathogen infection. Its functional output is not determined solely by its abundance but is shaped by cellular conditions and functional state, which are influenced by post-translational modifications, subcellular localization, molecular interactions, metabolic conditions, and extracellular or pathogen-derived stimuli. In this review, we summarize current evidence for the context-dependent functions of host ENO1 during infection, with particular emphasis on its roles in metabolic reprogramming, regulation of gene expression and pathogen replication, and modulation of host immunity and inflammation. We further discuss how pathogens can manipulate host ENO1 expression, stability, localization, or molecular interactions to facilitate infection, and distinguish these functions from those of pathogen-derived enolases, which contribute to microbial metabolism, invasion, and host interaction. By integrating these findings, we propose that ENO1 functions as a multifunctional regulatory hub, with its biological activities emerging from the specific molecular and cellular environments in which it operates. This framework provides a mechanistic perspective for understanding how a single conserved protein can engage distinct molecular partners and biological processes across different cellular and infectious settings, highlighting the functional versatility of ENO1 at the host–pathogen interface.

## 1 Introduction

ENO1 (α-enolase) is a highly conserved member of the enolase family that catalyzes the reversible dehydration of 2-phosphoglycerate (2-PG) to phosphoenolpyruvate (PEP), representing the penultimate step of glycolysis. As an essential component of glycolytic metabolism, enolase proteins are broadly distributed across bacteria, fungi, protozoa, plants, and animals, highlighting the ancient evolutionary origin and wide distribution of this metabolic enzyme (1, 2). In mammals, ENO1, the α-enolase isoform encoded by the *ENO1* gene, was regarded primarily as a housekeeping enzyme supporting cellular energy production. However, accumulating evidence has demonstrated that ENO1 participates in diverse biological processes beyond glycolysis. In addition to its metabolic activity in the cytoplasm, intracellular ENO1 can participate in transcriptional and post-transcriptional regulation through its interactions with nucleic acids and regulatory proteins (3, 4). At the cell surface, ENO1 functions as a plasminogen receptor and contributes to extracellular matrix (ECM) remodeling, cell migration, and tissue invasion (5, 6). Extracellular ENO1 has also been implicated in the regulation of inflammatory and immune responses (7–10). Collectively, these findings have expanded the functional landscape of ENO1, establishing it as a multifunctional protein that links cellular metabolism with gene regulation, extracellular signaling, and diverse cellular responses.

Importantly, the functional diversity of ENO1 is unlikely to represent a collection of independent “moonlighting” activities. Rather, its diverse functions are interconnected and shaped by multiple regulatory inputs, including metabolic state, cellular and environmental stimuli, post-translational modifications (PTMs), subcellular localization, and molecular interactions. Changes in these inputs can alter the functional state of ENO1 and redirect its activity toward distinct cellular processes, linking metabolism with gene regulation, signaling, and immune responses. Thus, ENO1 can be viewed not simply as a multifunctional protein, but as a multifunctional hub that integrates metabolic and regulatory signals to coordinate cellular responses.

This hub-like property is particularly relevant at the host–pathogen interface, where metabolic remodeling, immune regulation, and pathogen replication are closely interconnected. Infection can alter ENO1 abundance, PTMs, localization, and molecular interactions, while ENO1-mediated metabolic and non-metabolic activities can in turn influence pathogen replication, innate immune signaling, inflammation, and tissue responses (11–15). For example, ENO1-mediated metabolic changes can reshape immune cell functions, providing a mechanistic link between its metabolic activity and host defense (16). Conversely, pathogens may manipulate ENO1-associated processes to support their replication or evade host responses (7, 11, 17–19). These bidirectional interactions illustrate how ENO1 can connect metabolic and immune processes rather than functioning through isolated activities.

The multifunctional nature of enolase is also evident in pathogen-derived enolases, which, beyond their conserved metabolic role, can participate in host adhesion, invasion, immune modulation, and pathogen fitness (20–23). Although these proteins differ from host ENO1 in their regulatory contexts and biological functions, their functional diversification further highlights the versatility of the enolase family at the host–pathogen interface.

Despite increasing evidence linking enolases to infection and immunity, current knowledge remains fragmented across metabolism, microbiology, immunology, parasitology, and related fields. Host ENO1 is often described either as a conserved glycolytic enzyme or as a multifunctional protein with seemingly unrelated activities, while pathogen-derived enolases are frequently considered separately as microbial virulence-associated factors. An integrated view of how metabolic states, PTMs, localization, and molecular interactions coordinate the diverse functions of ENO1 during infection is still lacking. In this review, we therefore discuss the regulatory basis of ENO1 functional plasticity and its roles in metabolism, gene regulation, pathogen replication, and host immunity, with particular emphasis on how these activities are interconnected. We further consider pathogen-derived enolases and emerging therapeutic approaches targeting enolase-associated pathways. By viewing ENO1 as a multifunctional hub, this review aims to provide an integrated perspective on how a conserved metabolic enzyme can participate in complex host–pathogen interactions and broader cellular regulation.

## 2 Molecular architecture and regulatory logic of ENO1

### 2.1 Molecular architecture, evolutionary conservation, and regulatory diversity of ENO1

Mammalian cells express three major enolase isoforms (α, β, and γ), encoded by *ENO1*, *ENO3*, and *ENO2*, respectively. Among them, α-enolase is encoded by *ENO1* and is broadly expressed across tissues. Enolase is a dimeric metalloenzyme, and functional complexes can form as homo- or heterodimers, including αα, αβ, ββ, αγ, and γγ, with their distribution varying among tissues and developmental stages (24).

Phylogenetic analysis based on representative enolase amino-acid sequences revealed a clear separation between eukaryotic and prokaryotic proteins, with the two groups forming distinct major evolutionary lineages. Within the eukaryotic lineage, vertebrate enolase proteins clustered relatively closely, with mammalian sequences forming a compact subclade, whereas avian, amphibian, and teleost proteins occupied more divergent positions (**Figure 1**). Fungal and protozoan enolases formed distinct branches, reflecting further lineage-specific diversification among eukaryotic organisms. Among prokaryotes, bacterial enolases formed a separate cluster, whereas the archaeal enolase from *Methanocaldococcus jannaschii* occupied a more distant position (**Figure 1**). Comparison of the selected amino-acid sequences revealed substantial conservation within both eukaryotic and prokaryotic groups, with pairwise sequence identities ranging from 43.2% to 97.3% and 49.2% to 93.6%, respectively. Despite evolutionary divergence among species, the overall conservation of enolase sequences is consistent with strong evolutionary constraints imposed by its essential role in glycolysis and energy metabolism. Meanwhile, the divergence observed among distinct evolutionary lineages suggests that enolase proteins have undergone lineage-specific functional specialization, potentially contributing to differences in their biological functions beyond their conserved metabolic role.

**Figure 1:**
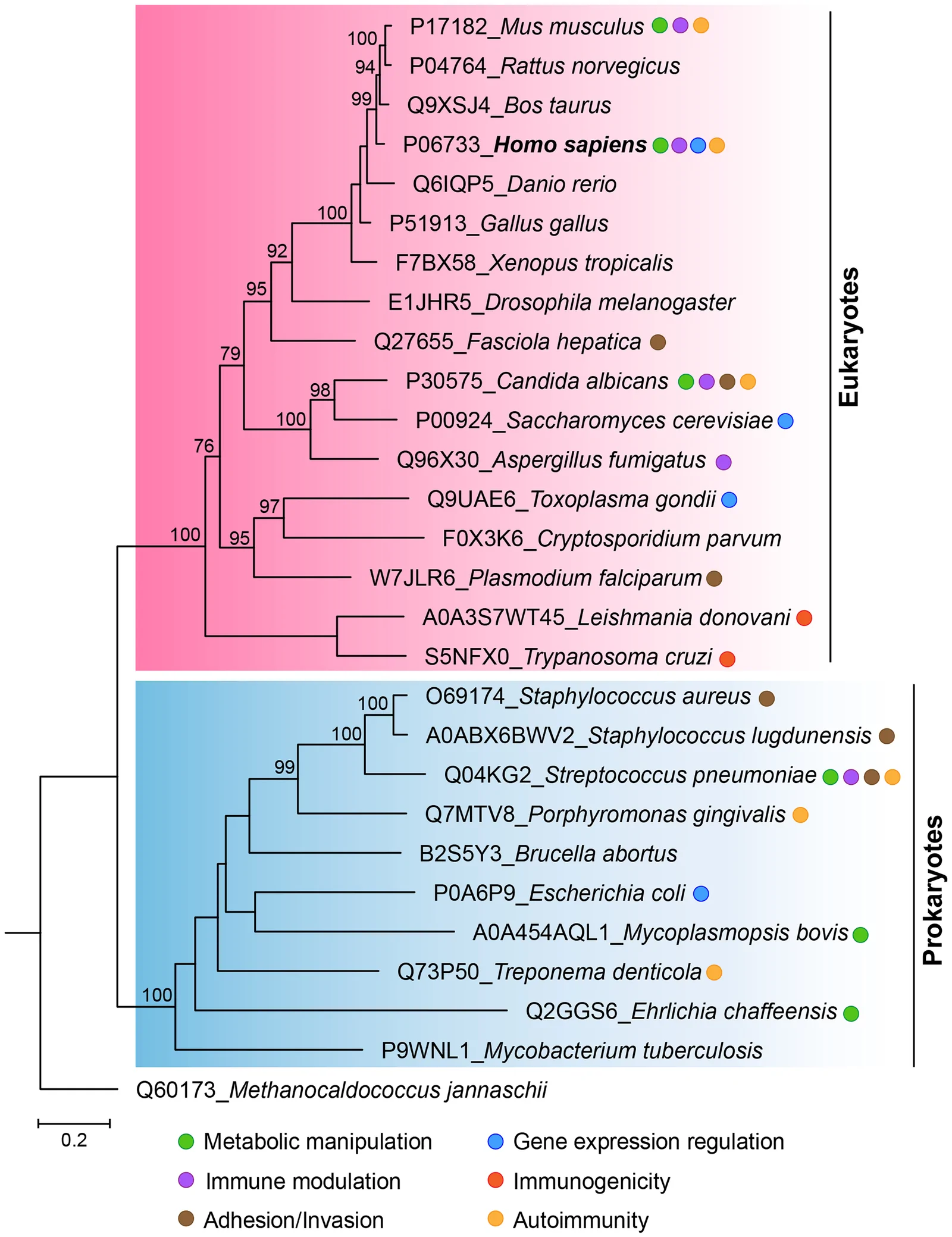
Phylogenetic relationships and functional diversity of enolase 1 (ENO1) across different species. ENO1 amino acid sequences from representative species were retrieved from UniProt and used to construct a phylogenetic tree using the maximum-likelihood method implemented in IQ-TREE. The tree was visualized using FigTree, and bootstrap values higher than 70 are shown at the corresponding branches. *Methanocaldococcus jannaschii* was used as the outgroup to root the tree. Colored dots at each tip indicate the distinct functions of ENO1 described for the corresponding species in this review.

Structurally, α-enolase is composed of two major domains, an N-terminal small domain and a larger C-terminal catalytic domain. The C-terminal domain contains the principal catalytic architecture required for the conversion of 2-PG to PEP, whereas the N-terminal domain contributes to protein folding, dimerization, and interactions with other cellular components. Enolase activity requires divalent metal ions, particularly magnesium ions (Mg^2+^), which coordinate the substrate and catalytic residues to facilitate the dehydration reaction (25). The dimeric organization further contributes to structural stability and enzymatic activity (26, 27). Together, the modular architecture, catalytic metal-binding site, and capacity for dimerization provide a structural basis for the well-established glycolytic activity of ENO1 while also enabling interactions with diverse proteins and cellular compartments, thereby supporting its functions beyond glycolysis.

ENO1 expression is regulated at multiple levels. At the transcriptional level, hypoxia-inducible factor 1α (HIF-1α) can induce ENO1 expression under hypoxic conditions that frequently accompany inflammatory and infectious microenvironments. At the post-transcriptional level, several microRNAs, including miR-22-3p, miR-5194, and miR-125a, directly target the 3′ UTR of ENO1 mRNA and suppress its expression (28–30). Conversely, long noncoding RNAs (lncRNAs) can regulate ENO1 through miRNA-dependent and RNA modification-dependent mechanisms. For example, MIR4435-2HG promotes ENO1 expression by sponging miR-143-3p in the cytoplasm and enhancing ENO1 mRNA stability through NAT10-mediated ac4C modification in the nucleus (31). Circular RNAs (circRNAs) represent another layer of ENO1 regulation, acting through distinct mechanisms that include modulation of host-gene expression and direct control of ENO1 protein stability. CircRNA-ENO1 enhances ENO1 expression, whereas circRPN2 and circBARD1 interact directly with ENO1 protein to promote its degradation (32–34). ENO1 is also subject to epitranscriptomic regulation. N6-methyladenosine (m6A) modification mediated by the RBM15/METTL3 complex promotes ENO1 translation in a YTHDF1-dependent manner, and this pathway can be induced by TGF-β/Smad2/3 signaling (35). Other RNA modifications, including KIAA1429-associated m6A regulation and NAT10-mediated N4-acetylcytidine (ac4C) modification, have also been implicated in ENO1 mRNA stability or translation (36, 37).

In addition to full-length α-enolase, MBP-1 is produced from the *ENO1* transcript through alternative translation initiation. MBP-1 lacks the N-terminal region required for the full enzymatic function of α-enolase and localizes predominantly to the nucleus, where it has been reported to repress *MYC* transcription, linking *ENO1*-derived protein products to transcriptional and cell-cycle regulation (38).

### 2.2 Post-translational modifications orchestrate ENO1 functional plasticity

Post-translational modifications (PTMs) provide an important layer of ENO1 regulation by modulating its enzymatic activity, RNA-binding properties, protein interactions, subcellular localization, and immunological recognition. Rather than uniformly activating or inhibiting ENO1, distinct PTMs and modification sites can selectively modify specific functional properties, allowing ENO1 activity to be fine-tuned in response to metabolic and cellular signals.

PTMs can first modulate the metabolic activity of ENO1. PRMT5-mediated symmetric dimethylation of ENO1 at Arg9 promotes formation of catalytically active ENO1 homodimers, and this modification is enhanced under high-glucose conditions, thereby increasing glycolytic flux (39). Similarly, O-GlcNAcylation of ENO1 at Thr19 promotes active dimer formation and enhances glycolytic activity, linking glucose availability to ENO1 function (9). Conversely, UFL1-mediated UFMylation of ENO1 at K285 and K420 is critical for regulating ENO1 function, as it disrupts ENO1 dimerization and attenuates its enzymatic activity (27). Together, these findings indicate that PTMs can regulate ENO1 enzymatic activity by controlling its oligomeric state and catalytic efficiency.

Beyond its metabolic role, ENO1 can function as an RNA-binding protein, and PTMs can selectively modulate this non-canonical activity. Acetylation of ENO1 enhances its RNA-binding capacity and is associated with reduced glycolytic activity, providing a mechanism through which RNA–protein interactions can regulate *ENO1 metabolism* (40). Lactylation provides another example of PTM-dependent regulation of ENO1 RNA binding. In sepsis, P300-mediated lactylation of ENO1 at Lys71 (K71) reduces its binding to TRIM21 mRNA, resulting in increased TRIM21 expression and contributing to endothelial dysfunction (41). ENO1 K193 lactylation in pulmonary endothelial cells during acute respiratory distress syndrome (ARDS) illustrates this dual regulation. In addition to enhancing ENO1 catalytic activity and glycolytic flux, K193 lactylation weakens ENO1 binding to CXCL12 mRNA, thereby relieving translational repression and increasing CXCL12 production (42). Thus, the same modification can simultaneously enhance the canonical metabolic function of ENO1 while altering its RNA-binding activity. The distinct effects of K193 and K71 lactylation further illustrate the site specificity of ENO1 PTM regulation. Whereas K193 lactylation enhances catalytic activity while reducing binding to CXCL12 mRNA, K71 lactylation primarily affects ENO1 binding to TRIM21 mRNA. These observations suggest that PTMs do not simply switch ENO1 between an “active” and “inactive” state. Instead, modification at different residues may redistribute ENO1 among distinct functional states, in which its catalytic activity and RNA-binding properties are differentially regulated.

PTMs can also regulate ENO1 through mechanisms beyond catalytic activity and RNA binding. O-GlcNAcylation at Ser249 decreases the interaction between ENO1 and PD-L1, thereby reducing STUB1-mediated PD-L1 degradation and promoting immune evasion in colorectal cancer (9). Phosphorylation at Ser419 has been reported to promote cytoplasmic localization of ENO1 in triple-negative breast cancer cells, apparently by facilitating exportin-1/CRM1-mediated nuclear export (43). Citrullination provides a distinct example in which PTM alters the immunological recognition of ENO1. Conversion of arginine residues to citrulline generates epitopes recognized by anti-citrullinated α-enolase antibodies in rheumatoid arthritis (RA), establishing citrullinated ENO1 as an important autoantigenic target (44–46).

Collectively, these findings indicate that ENO1 PTMs constitute a multilayered regulatory system that connects metabolic status with the diverse functions of ENO1. Rather than exerting a uniform effect on the protein, individual modifications can selectively regulate catalytic activity, RNA binding, protein interactions, localization, or immunomodulatory activity. In particular, modifications such as K193 lactylation illustrate how a single PTM can coordinate ENO1’s metabolic and RNA-binding functions, providing a potential molecular mechanism through which changes in cellular metabolism can be transmitted to gene regulation and immune-related processes.

### 2.3 Spatial regulation of ENO1 function

Subcellular localization is an important determinant of ENO1 function because it defines the molecular environment in which ENO1 encounters substrates, nucleic acids, protein partners, and signaling molecules (**Figure 2**). Thus, the functional properties of ENO1 are not determined solely by its intrinsic biochemical activity, but also by where the protein is localized and which molecular interactions are available at a given site. This spatial regulation provides a basis for the functional diversification of ENO1 beyond its canonical role in glycolysis.

**Figure 2:**
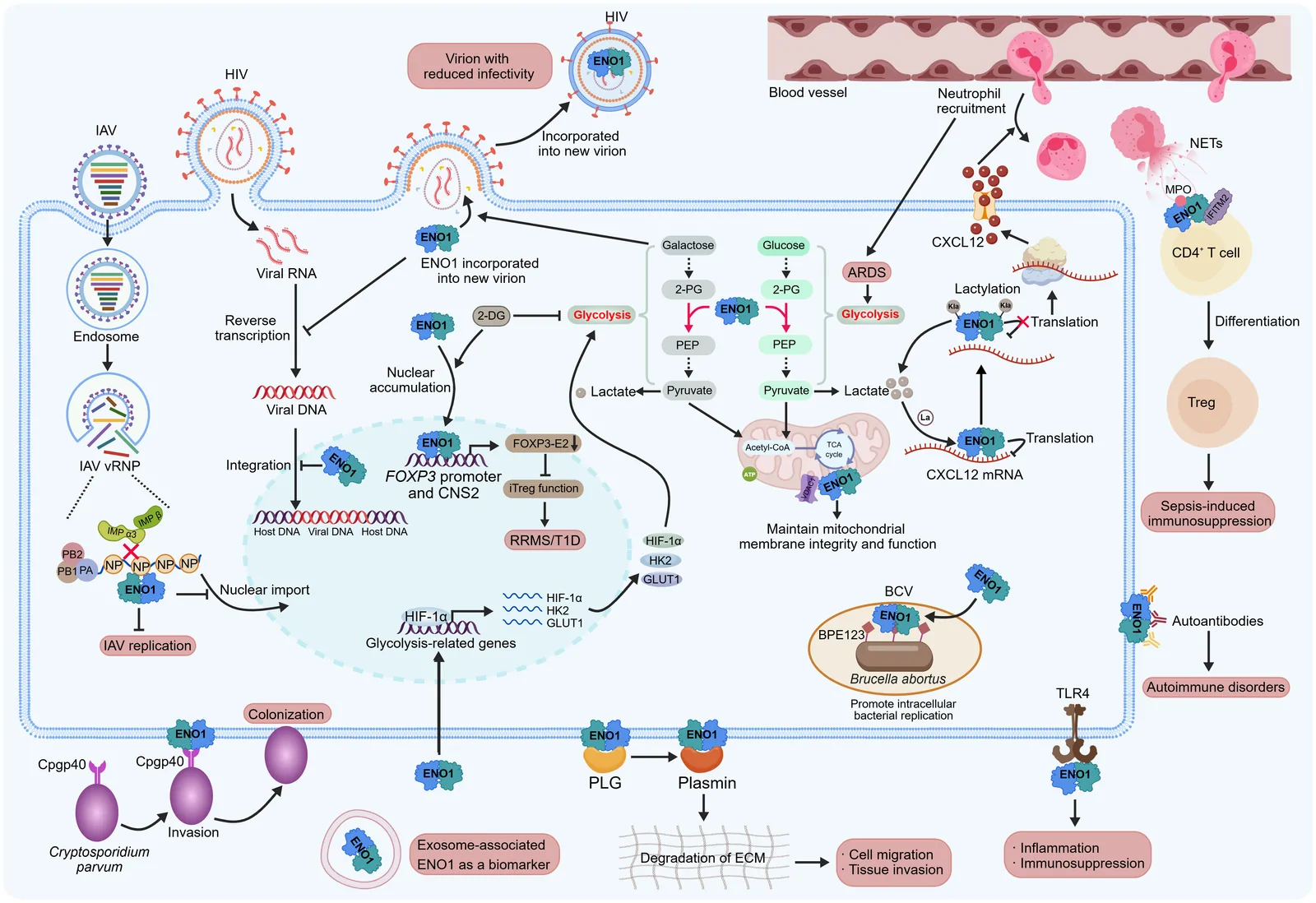
Context-dependent functional states of ENO1 at the host–pathogen interface. Various regulatory inputs, including post-translational modifications (PTMs), interacting partners, subcellular localization, metabolic states, and cellular or pathogen-derived stimuli, collectively shape the functional state of ENO1. Together, these regulatory inputs determine the molecular context in which ENO1 operates, thereby enabling its functional versatility across diverse biological processes. The figure was created with BioGDP (47).

In the cytoplasm, ENO1 primarily functions as a glycolytic enzyme, catalyzing the conversion of 2-PG to PEP and contributing to cellular energy metabolism. In contrast, nuclear localization places ENO1 in proximity to chromatin-associated factors and transcriptional regulators, enabling functions distinct from its cytoplasmic metabolic activity. For example, in regulatory T cells, nuclear ENO1 has been implicated in the repression of *FOXP3* transcription, linking its subcellular distribution to transcriptional regulation and immune tolerance (3). At the cell surface, ENO1 functions as a plasminogen receptor and can facilitate plasminogen activation, thereby contributing to extracellular matrix remodeling and cell migration (48). A fraction of ENO1 has also been detected in mitochondria in cardiomyocytes, where it interacts with VDAC1 and contributes to mitochondrial membrane integrity and function (49) (**Figure 2**). These examples illustrate that redistribution of ENO1 can expose the protein to distinct molecular partners and substrates, thereby enabling different functional outputs.

Extracellular localization further expands the functional repertoire of ENO1. ENO1 can also be released into the extracellular environment through non-classical secretory mechanisms and can associate with extracellular vesicles (EVs) (**Figure 2**). Extracellular ENO1 has been implicated in innate immune activation and inflammatory signaling, whereas EV-associated ENO1 has been investigated as a potential biomarker of disease progression (50–53). In addition, extracellular ENO1 can promote HIF-1α-associated glycolytic reprogramming and support disease-associated cellular phenotypes, further illustrating the functional versatility of ENO1 in the extracellular environment (54).

Importantly, subcellular localization does not act independently of other regulatory mechanisms. By determining which molecular partners are accessible to ENO1, localization can influence the composition and functional consequences of ENO1-containing molecular complexes. PTMs may further modulate this process by altering ENO1 localization, protein interactions, or binding properties. For example, phosphorylation at Ser419 has been reported to promote cytoplasmic localization of ENO1 by facilitating exportin-1/CRM1-mediated nuclear export (43). In this way, PTM-dependent changes in ENO1 localization may alter its access to compartment-specific interaction partners and consequently change its functional output.

Taken together, cytoplasmic, nuclear, membrane-associated, mitochondrial, and extracellular pools of ENO1 represent distinct functional states (**Figure 2**). The functional outcome of ENO1 is likely determined by the interplay between its localization, PTM state, and local molecular environment, which together define the molecular interactions and activities accessible to the protein. This integrated regulation provides a mechanistic basis for the functional versatility of ENO1, enabling changes in cellular context to be translated into distinct metabolic, signaling, transcriptional, and immune-related outputs.

## 3 Metabolic reprogramming in infection

Infection is frequently accompanied by profound changes in host cellular metabolism, as pathogens and infected cells remodel metabolic pathways to meet the energetic and biosynthetic demands of infection. One characteristic metabolic state is the Warburg effect, originally described in cancer cells, in which glucose is preferentially metabolized through aerobic glycolysis with increased lactate production despite the availability of oxygen (55, 56). Beyond cancer, similar metabolic adaptations have been observed during infection and are often referred to as Warburg-like metabolic reprogramming (57). Enhanced glycolytic flux in this state can provide not only ATP but also metabolic intermediates required for nucleotide, amino acid, and lipid biosynthesis, thereby reshaping the cellular environment in which pathogen replication occurs. Evidence for Warburg-like metabolic reprogramming has been reported during influenza A virus (IAV) infection. In A549 cells and mice infected with IAV, integrated metabolomic and transcriptomic analyses revealed enhanced glycolysis accompanied by increased expression of glycolytic regulators, including hexokinase 2 (HK2) and lactate dehydrogenase A (LDHA), together with elevated lactate levels. Increased glucose availability promoted viral replication, whereas inhibition of glycolysis reduced viral replication (58). These findings indicate that viral infection can induce a Warburg-like metabolic state that is closely linked to the metabolic requirements of pathogen replication. Among the enzymes involved in glycolysis, ENO1 catalyzes the conversion of 2-PG to PEP and may therefore participate in infection-associated metabolic reprogramming.

### 3.1 Host ENO1 supports pathogen replication through glycolytic reprogramming

Accumulating evidence suggests that host ENO1 can contribute to pathogen replication by participating in infection-associated glycolytic reprogramming. In HBV-associated HCC cells and mouse models, ENO1 expression is significantly upregulated, accompanied by enhanced glycolysis, increased glucose consumption, and elevated lactate production, consistent with a Warburg-like metabolic phenotype that creates a metabolic environment conducive to HBV replication (59). In cells infected with enterovirus A71 (EV-A71), the expression level of ENO1 and glycolytic activity was increased. ENO1 depletion or pharmacological inhibition of glycolysis impaired viral replication (60). These findings suggest that EV-A71 may exploit enhanced host glycolytic metabolism, at least in part through increased ENO1 abundance, to support efficient viral replication. Consistent with this interpretation, treatment with an anti-ENO1 monoclonal antibody reduced EV-A71 infection, viral burden, and neuropathology *in vivo*, supporting ENO1 as a potential host-directed therapeutic target (61).

### 3.2 Metabolic state can impose constraints on pathogen fitness

Importantly, infection-associated metabolic reprogramming does not invariably benefit pathogen replication. Rather, changes in cellular metabolism can influence pathogen fitness by altering the availability of metabolic substrates, host cellular pathways, and the molecular composition of pathogen progeny. This concept is illustrated by studies of human immunodeficiency virus type 1 (HIV-1) infection in CD4^+^ T cells. Although aerobic glycolysis supports efficient viral production, the metabolic state of infected cells can also influence the composition and infectivity of newly produced virions. When infected cells are shifted toward oxidative metabolism by replacing glucose with galactose in the culture medium, glycolytic enzymes, including ENO1, are more abundantly incorporated into nascent virions, and this altered virion composition is associated with reduced viral infectivity (62) (**Figure 2**). These findings indicate that cellular metabolic state can influence the molecular composition and functional quality of progeny virions, thereby imposing constraints on viral fitness.

Taken together, these findings indicate that the relationship between ENO1-associated glycolysis and pathogen infection is not simply determined by the overall level of glycolytic activity. Instead, the effects of ENO1 and cellular metabolism on pathogen replication are shaped by the pathogen, host cell, and stage of infection. ENO1 may therefore serve as a link between cellular metabolic state and pathogen fitness, while its broader cellular functions further expand the ways in which metabolic and non-metabolic processes can intersect during infection.

Besides, the consequences of ENO1-associated metabolic remodeling may differ among cell types during infection. Enhanced glycolysis may support antimicrobial effector functions in certain immune cells, whereas increased glycolytic activity in infected cells may provide metabolic resources that favor pathogen replication. Thus, ENO1-mediated metabolic regulation may contribute to a dynamic metabolic competition between host defense and pathogen demands.

## 4 ENO1-mediated regulation of gene expression and viral replication

Beyond its canonical role in glycolysis, ENO1 participates in the regulation of gene expression and viral replication through interactions with DNA, RNA, and protein partners. These functions are closely linked to the dynamic subcellular localization of ENO1 and its regulation by post-translational modifications and cellular metabolic states. In particular, nuclear accumulation can expose ENO1 to transcriptional and chromatin-associated regulatory processes, whereas interactions with cellular or viral RNAs and proteins can redirect ENO1 toward post-transcriptional regulation or pathogen-associated processes. Thus, rather than representing isolated non-glycolytic activities, these functions illustrate how ENO1 can act as a molecular interface connecting metabolic status with transcriptional, post-transcriptional, and host–pathogen regulatory networks.

### 4.1 DNA-associated regulation of gene expression

The ability of ENO1 to influence gene expression was initially linked to MBP-1, an alternatively translated product of the *ENO1* gene. MBP-1 binds the *c-myc* P2 promoter immediately upstream of the TATA box and represses *c-myc* transcription, thereby antagonizing oncogenic signaling pathways, including Notch1-mediated activation of c-myc (38, 63, 64). These findings established an early link between the ENO1 locus and transcriptional regulation.

Subsequent studies have shown that full-length ENO1 can also accumulate in the nucleus and participate in DNA-associated regulation. In the testicular microenvironment, nuclear ENO1 binds to the distal region of the *Elmo1* promoter and promotes *Elmo1* transcription, thereby facilitating apoptotic cell clearance and limiting inflammation (65). In human T cells, metabolic state can also influence ENO1-dependent transcriptional regulation. In conventional T cells, inhibition of glycolysis with 2-deoxy-D-glucose (2-DG), a glucose analog and glycolysis inhibitor, impaired the suppressive function of induced regulatory T cells, contributing to loss of immune tolerance. Mechanistically, glycolytic inhibition was associated with increased recruitment of ENO1 to the *FOXP3* promoter and conserved noncoding sequence 2 (CNS2), where nuclear ENO1 repressed FOXP3-E2 expression. Consistent with these findings, T cells from patients with relapsing-remitting multiple sclerosis (RRMS) or type 1 diabetes (T1D) exhibited impaired glycolysis accompanied by reduced FOXP3-E2 expression and diminished suppressive function of induced regulatory T cells (iTreg cells) (3). These observations suggest that nuclear ENO1 can couple cellular metabolic status to transcriptional programs governing immune homeostasis.

### 4.2 RNA-associated regulation of gene expression

In addition to DNA-associated functions, ENO1 can interact with RNA and participate in RNA-associated regulatory processes. Early studies in yeast demonstrated a role for enolase in mitochondrial tRNA import, whereas subsequent studies identified ENO1 as an RNA-binding protein capable of recognizing specific RNA sequences, including CUG repeat-containing RNAs (66, 67). More recent studies have expanded the repertoire of ENO1-associated RNAs to include mRNAs, lncRNAs, and circRNAs, suggesting that RNA interactions provide another layer through which ENO1 can regulate gene expression and cellular signaling (42, 68, 69). These RNA-associated functions can occur through distinct regulatory mechanisms. In cardiac fibroblasts, the lncRNA CFIRL recruits ENO1 to the *IL-6* promoter, where ENO1 acts as a transcriptional activator to enhance IL-6 expression and promote cardiac remodeling (70). In another regulatory mode, circFndc3b interacts with ENO1 and enhances the association of ENO1 with the 3′ untranslated region of Klf2 mRNA, increasing Klf2 mRNA stability and suppressing NLRP3 inflammasome-mediated pyroptosis (4). Similarly, circNFKB1 associates with ENO1 and promotes NFKB1 expression, thereby sustaining NF-κB signaling in osteoarthritic chondrocytes (8). In lung cancer, circFUT8 directly interacts with ENO1 and stabilizes its protein expression, enhancing glycolytic activity and promoting M2 macrophage polarization and tumor progression (71).

Collectively, these studies indicate that RNA-associated interactions can modulate multiple properties of ENO1, including its localization, stability, RNA-binding activity, and transcriptional functions. Conversely, RNA molecules can also use ENO1 as an interaction partner or regulatory effector to influence the expression or stability of other genes. This bidirectional relationship further expands the functional network centered on ENO1, linking RNA-mediated regulation with metabolism, inflammation, and immune responses. The functional consequences of ENO1–RNA interactions are likely to depend not only on the identity of the interacting RNA but also on the cellular compartment, metabolic state, and molecular modifications that shape ENO1 availability and activity.

### 4.3 Direct interaction with viral replication machinery

ENO1 can also directly interact with viral proteins and replication-associated complexes, thereby influencing viral gene expression and replication independently of its canonical glycolytic activity. During Sendai virus (SeV) infection, ENO1 functions as a host cofactor for viral transcription. Together with phosphoglycerate kinase (PGK) and tubulin, ENO1 enhances viral mRNA synthesis, suggesting that host metabolic proteins can be recruited to support paramyxovirus transcription (14).

In contrast, ENO1 has been described as an antiviral factor against several viruses. During HIV-1 infection, ENO1 can restrict infection at multiple stages of the viral life cycle. In virus-producing cells, virion-associated ENO1 suppresses early reverse transcription in newly infected target cells, thereby reducing the infectivity of progeny virions (72). In target cells, nuclear ENO1 further restricts infection by interfering with viral genome integration (73) (**Figure 2**). These findings highlight how the antiviral activity of ENO1 can arise from distinct cellular pools and occur at different stages of viral infection.

Evidence from IAV infection further illustrates the ability of ENO1 to directly engage viral replication machinery. Early studies identified associations between ENO1 and the viral nucleoprotein (NP) and matrix protein 1 (M1), suggesting that ENO1 may participate in processes involving viral ribonucleoprotein (vRNP) organization or trafficking (74, 75). Subsequent mechanistic studies demonstrated that ENO1 negatively regulates IAV replication by interacting with NP, disrupting the association between NP and importin-α3, and consequently impairing nuclear import of vRNP complexes. This activity suppresses viral polymerase function and viral replication independently of the glycolytic activity of ENO1 (76) (**Figure 2**).

These observations illustrate an important principle: the contribution of ENO1 to viral infection cannot be predicted simply from its canonical metabolic function. Instead, viral infection can engage distinct ENO1 pools and interaction networks. The outcome is shaped by the identity of the virus, the infected cell type, the subcellular localization of ENO1, its molecular modification state, and the stage of the viral life cycle.

## 5 ENO1 as a coordinator of host immunity

Beyond its canonical role in glycolysis and its direct interactions with viral replication machinery, ENO1 participates in multiple layers of host immune regulation. These functions are closely connected to the regulatory principles described above, including changes in metabolic state, PTM, subcellular localization, and molecular interactions. Through these regulatory layers, ENO1 can shift between metabolic, intracellular signaling, and extracellular functions, allowing it to influence antiviral responses, inflammatory activation, immune-cell function, and tissue responses. Thus, ENO1 represents a functional interface through which metabolic and cellular signals can be integrated into specific immune responses.

### 5.1 Modulation of antiviral signaling

ENO1 can modulate innate antiviral signaling. During hepatitis B virus (HBV) infection, ENO1 expression is increased and is associated with enhanced viral replication and suppression of type I interferon (IFN-I) responses. Silencing ENO1 increases IFN-I production and the expression of interferon-stimulated genes (ISGs), supporting a role for ENO1 as a negative regulator of antiviral interferon signaling during HBV infection (7). Evidence from SARS-CoV-2 infection further suggests an association between ENO1 and inflammatory signaling. ENO1 expression is increased in infected cells together with components of the NF-κB-associated signaling network, including p50, p65, and TBK1 (77).

### 5.2 Metabolic control of immune effector functions

The metabolic activity of ENO1 provides another major route through which it regulates immunity. As discussed above, metabolic state can influence ENO1 abundance, localization, and functional interactions; conversely, ENO1-dependent glycolysis can shape the metabolic state of immune cells. In naïve T cells, relatively low glycolytic activity is associated with a quiescent state, whereas T-cell activation induces glycolytic flux to support biosynthesis, proliferation, and effector differentiation, with ENO1 contributing to this metabolic transition (16).

ENO1-dependent metabolic regulation also influences innate immune cells. In microglia and macrophages, increased glycolytic activity associated with ENO1 supports proinflammatory activation during neuroinflammation (78). In sepsis, ENO1 interaction with IL1R2 regulates glycolysis-dependent pyroptosis in macrophages, whereas Neutrophil Extracellular Traps (NETs)-associated ENO1 has been implicated in Treg differentiation through IFITM2 recruitment (10, 79) (**Figure 2**). In dendritic cells responding to *Chlamydia* infection, ENO1 deficiency impairs glycolytic pyruvate production and disrupts mitochondrial homeostasis through altered TOM20 and ANT regulation, compromising cell survival, maturation, and antigen-presenting capacity (80). In septic-induced coagulopathy, mouse Eno1 contributes to the maintenance of endothelial glycocalyx integrity through glycolytic regulation and is also associated with the infiltration of immune cells,, including T cells an macrophages (81). These findings support a model in which ENO1 links metabolic flux to immune-cell functional states rather than acting solely as a constitutive glycolytic enzyme (**Figure 2**).

Together, these findings indicate that ENO1-dependent metabolic regulation can influence immune-cell activation, survival, differentiation, and effector function across diverse cell types. Importantly, these effects illustrate the bidirectional relationship between metabolism and immunity: metabolic changes can alter the functional state of ENO1, while ENO1-mediated metabolic flux can in turn shape immune-cell behavior.

### 5.3 Surface and extracellular ENO1 in inflammation and tissue responses

In addition to its intracellular functions, ENO1 can translocate to the cell surface and extracellular space, where it acquires functions distinct from its canonical glycolytic activity. Surface-localized ENO1 acts as a plasminogen receptor and can facilitate localized proteolytic activity, thereby promoting ECM remodeling and the migration of neutrophils and macrophages into inflamed tissues (48, 82). Intracellular and surface-associated ENO1 has also been implicated in inflammatory signaling through pathways such as NF-κB and MAPK, contributing to the production of proinflammatory cytokines (83, 84). ENO1 has additionally been linked to IL-32 production in RA and to the regulation of pyroptosis during sepsis (85). Following IL-1β stimulation, ENO1 interacts with CRLF1 and promotes NF-κB signaling, contributing to osteoarthritis pathogenesis (86). Extracellular ENO1 can further function as an immune-signaling molecule. ENO1 is released through non-classical secretory mechanisms, including EV-associated and autophagy-dependent pathways, and can be detected in body fluids under pathological conditions (50, 51). Extracellular ENO1 has been proposed to function as a damage-associated molecular pattern (DAMP) that activates innate immune signaling through pattern-recognition pathways. In RA, extracellular ENO1 interacts with monocytes through a CD14-dependent TLR4 pathway, inducing an early proinflammatory response followed by delayed IL-10 production (53) (**Figure 2**). In glioblastoma, autophagy-dependent secretion of ENO1 activates TLR4-associated PI3K/Akt and ERK/SPHK1 signaling, while downstream sphingosine-1-phosphate (S1P) signaling promotes M2-like polarization of tumor-associated macrophages and contributes to an immunosuppressive microenvironment and chemoresistance (87).

Together, these findings indicate that surface and extracellular ENO1 can connect cellular stress and metabolic alterations to inflammatory signaling, leukocyte recruitment, tissue remodeling, and immune-cell polarization. And changes in ENO1 localization can effectively rewire its functional output.

### 5.4 ENO1 at the infection–autoimmunity interface

Changes in ENO1 regulation can also influence adaptive immune recognition. Under chronic inflammatory conditions, ENO1 can become a target of adaptive immunity and has emerged as an autoantigen in multiple autoimmune diseases. PTMs, particularly citrullination, can alter the antigenicity of ENO1 and contribute to the breakdown of immune tolerance (19, 44). Anti-ENO1 autoantibodies have been detected in RA, systemic lupus erythematosus (SLE), autoimmune retinopathy, and other autoimmune disorders, implicating ENO1 in the development or persistence of pathogenic immune responses (88–90).

The relationship between infection and ENO1 autoimmunity is particularly evident in RA. Citrullinated ENO1 is a major autoantigenic target associated with autoreactive immune responses, and anti-ENO1 antibodies can interact with aberrantly expressed surface ENO1 on monocytes and macrophages, promoting proinflammatory mediator production and amplifying synovial inflammation (44, 83, 91, 92). Importantly, the periodontal pathogen *Porphyromonas gingivalis* can modify host ENO1 through sequential proteolysis and citrullination. Its arginine-specific gingipain (rgp) first cleave ENO1 at Arg–X peptide bonds, generating peptides with C-terminal arginine residues that can subsequently be converted to citrulline by the bacterial peptidylarginine deiminase (ppad). This sequential modification generates ENO1-derived neoepitopes with altered antigenicity and may facilitate loss of immune tolerance and activation of autoreactive immune responses (19) (**Figure 3**). These observations place ENO1 at a potential interface between infection, chronic inflammation, and autoimmunity. Collectively, the diverse immune functions of ENO1 reflect its functional plasticity rather than independent activities. Changes in metabolic state and PTMs can alter ENO1 abundance, localization, and molecular interactions, thereby redirecting the same protein between glycolytic metabolism, intracellular signaling, surface-associated processes, extracellular immune regulation, and adaptive immune recognition. ENO1 can thus integrate metabolic and cellular stress and translate them into context-dependent immune outcomes, functioning as a metabolic–immune hub. Given its central position within these interconnected regulatory networks, ENO1 may also represent a strategic target for pathogens seeking to reshape the host cellular environment. This provides a rationale for considering how viruses and other pathogens manipulate host ENO1 to facilitate infection and evade or modulate host defenses.

**Figure 3:**
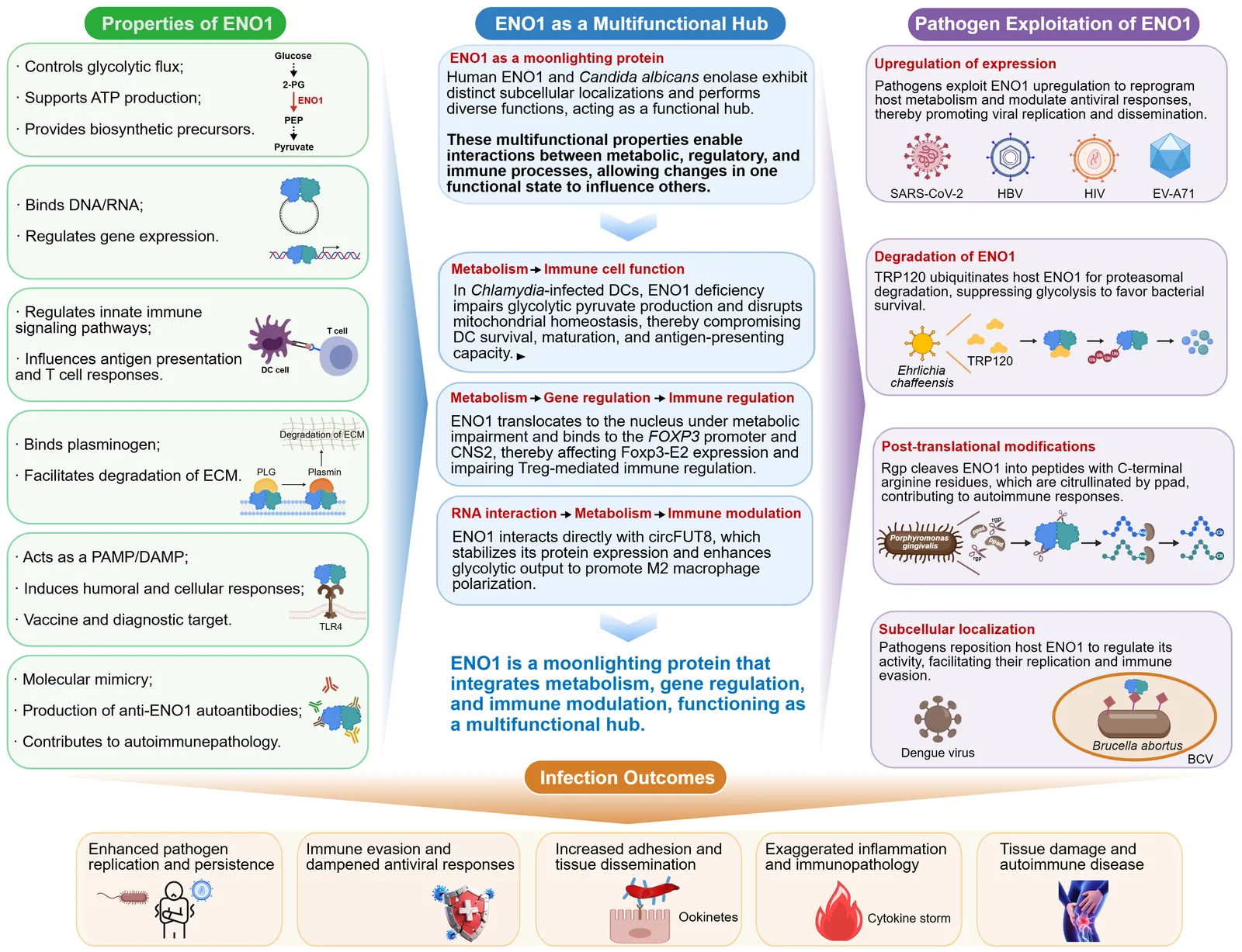
ENO1 as a multifunctional hub in host–pathogen interactions. Schematic illustration of the multifaceted roles of ENO1 and its function as a central molecular hub. ENO1 participates in diverse biological processes, including glycolysis, DNA/RNA binding, immune regulation, plasminogen-mediated invasion, immunogenicity, and autoimmunity. These diverse functions and properties enable ENO1 to serve as a functional hub that can be exploited by pathogens, thereby contributing to pathogen survival, invasion, replication, dissemination. The figure was created with BioGDP (47).

## 6 Pathogen manipulation of host ENO1

The regulatory plasticity of ENO1 described above also provides opportunities for pathogens to actively reprogram its abundance, stability, and subcellular distribution during infection. Rather than simply altering host metabolism as a downstream consequence of infection, pathogens can directly target ENO1 through pathogen-encoded factors or infection-associated processes. These interactions can redirect ENO1 toward cellular functions that favor pathogen persistence, replication, invasion, or dissemination. The following sections summarize two major strategies by which pathogens manipulate host ENO1: regulation of its expression and stability, and alteration of its subcellular localization.

### 6.1 Pathogen regulation of host ENO1 expression and stability

Accumulating evidence indicates that pathogens can directly regulate host ENO1 abundance and protein stability. Such regulation can alter ENO1-dependent metabolic activity and cellular functions, thereby modifying the host environment during infection. A representative example is HIV-1, which uses its accessory protein Nef to increase ENO1 expression in monocytes and macrophages. Increased ENO1 is associated with enhanced cellular migratory capacity and may facilitate viral dissemination across mucosal barriers (17). In contrast, the obligate intracellular bacterium *Ehrlichia chaffeensis* employs the effector protein TRP120, an E3 ubiquitin ligase mimic, to promote ubiquitination and proteasomal degradation of host ENO1. Loss of ENO1 reduces host glycolytic activity and is associated with enhanced bacterial intracellular survival and replication (18).

### 6.2 Pathogen hijacking of host ENO1 localization

Beyond regulating ENO1 abundance, pathogens can also redirect its subcellular localization, thereby placing ENO1 in cellular compartments where its functions can be exploited by the pathogen. In *Brucella abortus* infection, the type IV secretion system effector BPE123 interacts with host ENO1 and recruits it to the *Brucella*-containing vacuole (BCV). This interaction enhances ENO1 activity and promotes intracellular bacterial replication, indicating that host ENO1 can be actively incorporated into pathogen-containing replication niches (11) (**Figures 2**, **3**). Similarly, the protozoan parasite *Cryptosporidium parvum* exploits host ENO1 during invasion. Its sporozoite surface protein Cpgp40 interacts with ENO1, whereas functional inhibition of ENO1 by antibodies or siRNA reduces parasite invasion and intracellular development (12) (**Figure 2**). Pathogen-induced redistribution of ENO1 can also extend to the extracellular environment. Dengue virus infection promotes the release of ENO1 from hepatocytes and circulating ENO1 levels correlate positively with disease severity in infected patients (52).

Collectively, these studies suggest that pathogens exploit ENO1 not only by changing its abundance but also by redirecting it to pathogen-containing compartments or extracellular space. Spatial control of ENO1 may therefore represent an important mechanism through which pathogens selectively access its metabolic, signaling, or extracellular functions.

## 7 Pathogen-derived enolases at the host–pathogen interface

Although host ENO1 and pathogen-derived enolases share a conserved evolutionary and structural architecture, they constitute biologically distinct protein classes that operate within different cellular milieus and elicit divergent functional outcomes. Host ENO1, encoded by the host *ENO1* gene, serves as a multifunctional metabolic and regulatory hub that couples glycolysis to gene regulation, signaling, immune responses, and pathogen-associated processes. By contrast, pathogen-derived enolases, encoded by microbial genomes, support pathogen survival and fitness while also acquiring niche-specific roles in host colonization, tissue invasion, immune modulation, and microbial adaptation. Thus, the conserved enolase scaffold furnishes a shared structural foundation, whereas differences in localization, molecular interactions, and host biological settings enable pathogen-derived enolases to undergo functional diversification.

### 7.1 Metabolic functions of pathogen-derived enolases

Similar to host ENO1, pathogen-derived enolases retain their canonical glycolytic function, which supports microbial energy metabolism and survival. In *Candida albicans*, fungal Eno1 supports glycolytic metabolism and cellular viability, whereas baicalein-mediated inhibition of Eno1 enzymatic activity has been reported to impair fungal growth (93). Similarly, 2-aminothiazole targeting *Mycobacterium tuberculosis* enolase disrupt glycolytic metabolism and reduce cellular ATP levels (94). These observations indicate that pathogen-derived enolases represent important determinants of microbial metabolic fitness and survival.

### 7.2 Pathogen-derived enolase as virulence factors

Pathogen-derived enolases can contribute to microbial virulence through functions that extend beyond their canonical metabolic activity. Surface-associated enolases are particularly well characterized as multifunctional host-interacting proteins. By binding ECM components and recruiting host plasminogen, enolases can couple microbial adhesion to extracellular proteolysis, thereby facilitating tissue penetration and dissemination. Surface-associated enolases from diverse pathogens interact with laminin while simultaneously recruiting plasminogen, promoting plasmin generation and ECM remodeling (6, 95–97). This mechanism, characterized in multiple infectious organisms, facilitates microbial adhesion, tissue penetration, and systemic dissemination. In *Staphylococcus aureus*, α-enolase functions as a laminin-binding adhesin that promotes bacterial attachment to host tissues (98). Enolase from *Staphylococcus lugdunensis* similarly contributes to laminin binding and plasminogen recruitment, facilitating colonization and invasion (99). In *Streptococcus pneumoniae*, surface α-enolase recruits human plasminogen through multiple binding motifs and promotes its conversion to plasmin, thereby enhancing bacterial dissemination and pathogenicity (97). Comparable functions have been described in the trematode *Fasciola hepatica*, whose tegument-associated enolase binds laminin and plasminogen and may facilitate parasite attachment and tissue penetration (100). In *Candida albicans*, cell-wall-associated Eno1 efficiently recruits host plasminogen and promotes its conversion to plasmin, enhancing epithelial invasion and systemic dissemination. Disruption of the Eno1–plasminogen interaction reduces fungal invasion, providing functional evidence for the importance of plasminogen recruitment in fungal virulence (22). Enolase expressed on *Plasmodium ookinetes* also contributes to invasion of the mosquito midgut by interacting with midgut receptors and recruiting plasminogen, whereas antibodies against enolase can inhibit parasite growth and invasion (15, 101, 102).

Across these pathogens, surface-associated enolase therefore acts as a functional interface between the microbial cell and the host extracellular environment, linking a conserved metabolic protein to adhesion, proteolysis, tissue invasion, and dissemination. Importantly, pathogen-derived enolases can also participate in microbial regulatory processes that are independent of their catalytic activity. In pathogenic *Escherichia coli*, the small RNA DicF represses the cell-division gene ftsZ. Under anaerobic conditions associated with intestinal colonization, enolase interacts with RNase E and stabilizes DicF, thereby suppressing ftsZ expression and promoting bacterial filamentation. This morphological transition has been associated with enhanced bacterial persistence, immune evasion, and antibiotic tolerance (103). In *Toxoplasma gondii*, genetic deletion of TgENO1 causes widespread changes in gene expression, suggesting that TgENO1 may contribute to gene regulatory processes within the parasite (104).

These findings illustrate that pathogen-derived enolases can occupy multiple functional positions within microbial regulatory and host–pathogen networks. Rather than acting solely as glycolytic enzymes, they can connect central metabolism with microbial regulation, adhesion, invasion, persistence, and host interaction. Such functional plasticity provides a mechanistic basis for the broad contribution of pathogen-derived enolases to virulence.

### 7.3 Pathogen-derived enolase in host immune modulation

Beyond their roles in microbial metabolism and virulence, pathogen-derived enolases have emerged as important regulators of host immune responses. Unlike host ENO1, which mainly influences immunity through intracellular metabolic regulation, subcellular redistribution, and interactions with host signaling pathways, pathogen-derived enolases can directly interact with immune cells, complement components, and extracellular host molecules. Therefore, pathogen enolases represent a distinct class of microbial factors that can both activate host immunity and promote immune adaptation during infection.

Several studies have demonstrated that pathogen-derived enolases can stimulate host immune responses. Secreted Eno1 from *Candida albicans* promotes B-cell activation, antibody production, and IL-6 release, indicating that fungal enolase can function as an extracellular immunomodulatory molecule (105). Similarly, α-enolase from *Streptococcus pneumoniae* promotes neutrophil migration and NETs formation, demonstrating that bacterial enolases can actively participate in innate immune activation (21). These findings suggest that pathogen-derived enolases can actively shape immune cell responses.

In contrast, pathogen-derived enolases can also suppress host immune defense mechanisms to facilitate microbial persistence. In *Aspergillus fumigatus*, surface-associated AfEno1 recruits complement regulatory proteins, including factor H, factor H-like protein 1 (FHL-1), and C4-binding protein (C4BP), thereby promoting inactivation of C3b and C4b and limiting complement activation. At the same time, AfEno1-mediated plasminogen recruitment may further couple immune evasion with enhanced tissue invasion (106).

Pathogen-derived enolases may also modulate host inflammatory responses by reprogramming cellular metabolism. Secreted α-enolase from Mycoplasmopsis bovis interacts with host β-actin (ACTB), promoting glycolytic reprogramming and reactive oxygen species (ROS)-associated inflammatory signaling, including the HIF-1α/IL-1β and IL-6/TNF-α axes (13). These findings suggest that pathogen-derived enolases can influence host immunity not only through direct interactions with host molecules but also by coupling metabolic alterations to inflammatory responses.

Collectively, pathogen-derived enolases display considerable functional flexibility in host immune environments. They can stimulate inflammatory and innate immune responses while simultaneously exploiting complement and other host regulatory systems to facilitate microbial persistence. Their ability to engage both immune and metabolic processes further illustrates how a conserved metabolic protein can acquire distinct regulatory functions when positioned at the host–pathogen interface.

### 7.4 Evolutionary conservation, antigenicity, and molecular mimicry

#### 7.4.1 Antigenicity of pathogen-derived enolases

Beyond their metabolic and virulence-associated functions, pathogen-derived enolases can serve as prominent antigens during infection. Their extracellular or surface-associated localization in many pathogens provides opportunities for direct exposure to the host immune system, while their abundance and structural conservation may further contribute to their immunological visibility. Several studies have demonstrated that pathogen-derived enolases are recognized by both humoral and cellular immune responses. *Candida albicans* Eno1 is an immunogenic antigen during fungal infection, and antibodies against fungal enolase have been detected in infected individuals (107). Similarly, *Streptococcus pneumoniae* α-enolase exhibits strong immunogenicity and can be recognized by sera from patients with pneumococcal disease (96). In *Leishmania donovani*, enolase induces Th1-biased immune responses, further demonstrating that pathogen-derived enolases can actively participate in host immune recognition (108, 109). In addition, conserved T- and B-cell epitopes have been predicted within *Trypanosoma cruzi* enolase, suggesting that shared antigenic regions may represent common features among pathogen-derived enolases (110).

#### 7.4.2 Molecular mimicry and cross-reactivity

Microbial enolases share structural and sequence similarities with host ENO1, immune responses initially directed against pathogen-derived enolases may recognize homologous epitopes in host proteins. This phenomenon represents a form of molecular mimicry, in which pathogen-derived molecules resemble host components and potentially interfere with immune tolerance. Several studies have provided evidence supporting molecular mimicry involving pathogen-derived enolases. Antibodies against group A *Streptococcus enolase* can recognize both bacterial and human enolase, and cross-reactive antibodies have been detected in patients with acute rheumatic fever (111, 112). Similarly, *Treponema denticola* enolase exhibits substantial sequence similarity to human ENO1, and immunization with *Treponema denticola* enolase induces antibodies that cross-react with mouse Eno1 and is associated with increased TNF-α expression in gingival tissues (113). In patients with autoimmune polyglandular syndrome type 1 (APS-1), antibodies against *Candida albicans* Eno1 have also been detected, suggesting that fungal enolase may contribute to autoimmune responses through antigenic similarity with host ENO1 (114).

Molecular mimicry involving enolase-related epitopes is not restricted to microbial enolases. During viral infection, pathogen-derived proteins may also contain structural similarities with host ENO1-associated regions. In a rhesus rotavirus-induced model of biliary atresia, the viral surface protein VP4 contains sequence and structural similarities to ENO1, particularly within the immunogenic VP5 domain. Antibodies generated against rotavirus can cross-react with host ENO1, suggesting that structural similarity between a pathogen protein and ENO1-associated epitopes may contribute to infection-associated loss of self-tolerance and autoimmune pathology (115).

The antigenicity and functional versatility of pathogen-derived enolases also provide opportunities for therapeutic intervention. Several enolase-targeted strategies have been explored experimentally, including recombinant pathogen enolase vaccines and enolase-derived antigen peptides, anti-enolase antibodies, enzymatic inhibitors, RNA interference approaches, and inhibitors targeting pathogen-derived enolases. These strategies may interfere with pathogen metabolism, adhesion, invasion, or host–pathogen interactions, or enhance protective immune responses against infection. However, their therapeutic development is complicated by the high evolutionary conservation of enolases and the potential for cross-reactivity with host ENO1. **Table 1** summarizes representative enolase-targeted strategies, their potential therapeutic advantages, major limitations, and possible approaches for improving pathogen selectivity and therapeutic applicability.

**Table 1:** Reported ENO1-targeted molecules and approaches: therapeutic potential and limitations.

Targeting strategy	Targeted ENO1/enolase pool or mechanism	Representative molecules/approaches	Therapeutic potential	Advantages	Limitations	Strategies for improvement
Host-directed ENO1 inhibition (small-molecule inhibitors)	Intracellular host ENO1 catalytic activity; inhibition of glycolytic function	Pharmacological ENO1 inhibitors (e.g., glycolytic inhibitors targeting ENO1 enzymatic activity), including small molecules designed to target the ENO1 catalytic pocket, such as (116), SF2312 (117), and POMSF (118).	Potential suppression of the growth of certain cancer cells or the replication of pathogens by limiting ENO1-dependent metabolic support.	Directly targets the metabolic pathway exploited by cancer cells or pathogens	Host ENO1 is an essential glycolytic enzyme; systemic inhibition may cause metabolic toxicity; difficult to define therapeutic window between pathogen restriction and host metabolic impairment	Develop partial inhibitors, transient inhibition strategies, infection-site delivery systems, or compounds targeting infection-specific ENO1 states rather than basal glycolytic activity
Host ENO1 inhibition by monoclonal antibodies	Mainly cell-surface/extracellular ENO1 rather than intracellular glycolytic ENO1	In cancer therapy, anti-ENO1 monoclonal antibodies, such as 72/1 mAb (119) and chENO1-22 (120), have been reported to inhibit cancer cell invasion and metastasis. In infectious diseases, antibodies against ENO1 have been shown to block the invasion of *Cryptosporidium parvum* into host cells (12).	Potential inhibition of pathogen adhesion, invasion, or extracellular ENO1-mediated inflammatory functions; potential suppression of metastasis in certain cancer cells.	Greater selectivity toward extracellular ENO1 pools; avoids direct inhibition of intracellular glycolysis; can interfere with pathogen–host interaction interfaces	Antibodies have limited access to intracellular ENO1 and may interfere with physiological plasminogen activation, immune-cell migration, and tissue remodeling; some antibodies may also trigger inflammation or immune-mediated tissue damage.	Define antibody-accessible ENO1 epitopes; distinguish pathogen-exploited surface ENO1 functions from physiological ENO1 functions; develop infection-specific antibody targeting strategies
ENO1 expression suppression	Regulation of ENO1 abundance at transcriptional or post-transcriptional levels	siRNA/shRNA-mediated ENO1 depletion (12); regulatory RNA-based approaches	Reduction of ENO1 expression may suppress pathogen replication or inflammatory responses in ENO1-dependent settings	Allows direct assessment of ENO1 dependency; can reveal biological functions beyond glycolysis	Poor clinical feasibility for systemic delivery; broad reduction of ENO1 may affect normal metabolic homeostasis	Develop targeted delivery systems; combine with pathogen-specific targeting; exploit transient or localized suppression.
Targeting ENO1-associated molecular interactions	Non-canonical ENO1 functions mediated by protein/RNA interactions	Disruption of ENO1–viral protein interactions; inhibition of ENO1-associated RNA-binding or signaling complexes	May selectively block pathogen-supporting functions while preserving glycolytic activity	Potentially higher specificity than catalytic inhibition; enables separation of metabolic and non-metabolic functions	Interaction interfaces are complex and difficult to drug; limited structural information for some ENO1 complexes	Identify infection-specific ENO1 interactomes; develop interface-targeting molecules; apply structure-guided drug design.
Pathogen-selective enolase inhibition	Microbial enolases encoded by bacterial, fungal, or protozoan pathogens	Pathogen enolase inhibitors (e.g., 2-aminothiazole derivatives targeting Mycobacterium tuberculosis enolase (94); fungal enolase inhibitors such as baicalein targeting Candida albicans Eno1 (93)).	Direct inhibition of pathogen metabolism, survival, invasion, or virulence-associated functions	Avoids direct inhibition of host ENO1 if sufficient selectivity is achieved; targets pathogen-specific functions	High structural conservation between pathogen enolases and host ENO1 creates selectivity challenges; some inhibitors may also affect human ENO1	Identify pathogen-specific structural pockets; target non-conserved regulatory regions or extracellular virulence-associated functions; optimize pathogen-selective inhibitors.
Immunotherapeutic approaches against pathogen-derived enolases	Immune recognition of microbial enolase antigens	Recombinant pathogen enolase vaccines and enolase-derived antigen peptides (e.g., recombinant *Candida albicans enolase*) (121), which have shown protective effects against experimental candidiasis in mice.	Induction of protective immune responses against fungal, bacterial, or parasitic infections	Exploits strong immunogenicity of pathogen-derived enolases; may provide broad pathogen-targeting potential	Molecular mimicry risk due to the high conservation of pathogen enolase with host ENO1, with potential autoimmune responses requiring careful evaluation; moreover, immunogenicity does not necessarily translate into protective efficacy.	Select pathogen-specific epitopes; exclude conserved host-cross-reactive regions; evaluate long-term immune safety.

Collectively, the diverse functions of pathogen-derived enolases highlight the remarkable evolutionary conservation and functional plasticity of the enolase family. Across organisms ranging from microbes to mammals, enolases retain their essential role in central metabolism, underscoring their fundamental importance for energy production and cellular survival. At the same time, this conserved molecular framework has been adapted to support diverse regulatory and host-interacting functions, including modulation of RNA and gene expression, microbial adaptation, host metabolism, immune responses, adhesion, and tissue invasion. Thus, both host ENO1 and pathogen-derived enolases exhibit functional versatility beyond glycolysis, engaging in diverse regulatory processes that are shaped by their respective biological contexts. The same evolutionary relatedness, however, can also create molecular similarity between microbial enolases and host ENO1, providing a basis for molecular mimicry and immune cross-reactivity that may contribute to infection-associated autoimmune pathology. Taken together, these observations emphasize that enolase is not merely a conserved metabolic enzyme but a multifunctional hub across biological systems (**Figure 3**). Its conserved importance from microbes to mammals, together with its ability to connect metabolism, gene regulation, host interaction, and immunity, underscores the complexity of enolase function at the host–pathogen interface and the importance of understanding this protein family from both microbial and host perspectives.

## 8 Conclusions and future perspectives

ENO1 is increasingly recognized as a moonlighting protein whose biological significance extends far beyond its canonical role in glycolysis. Throughout this review, ENO1 has been implicated in metabolic regulation, transcriptional control, RNA-associated processes, signaling, immune responses, extracellular interactions, and the regulation of pathogen replication. These activities are shaped by its enzymatic state, PTMs, subcellular localization, and molecular interactions (**Figure 2**). Importantly, ENO1 should not be viewed simply as a protein with a collection of unrelated activities. Its metabolic and non-metabolic activities can influence one another, allowing ENO1 to couple metabolic changes with gene regulation, signaling, and immune responses. This functional interdependence provides a basis for considering ENO1 as a regulatory hub rather than merely a multifunctional protein (**Figure 3**).

Several findings illustrate this interconnected architecture. In dendritic cells responding to *Chlamydia* infection, ENO1 deficiency links impaired glycolytic pyruvate production to altered mitochondrial homeostasis and antigen-presenting capacity (80). Conversely, metabolic changes can modulate ENO1 binding to DNA and thereby couple cellular metabolism to the transcriptional regulation of *FOXP3* and downstream immune responses (3). ENO1 can also function in the opposite direction, in which an RNA-associated interaction influences its metabolic activity; for example, circFUT8 regulates ENO1 through RNA–protein interaction, resulting in altered glycolysis and subsequent immune regulation (71). Together, these examples demonstrate that ENO1-mediated metabolic, nucleic-acid-associated, and immune processes are functionally coupled. ENO1 can therefore serve not only as an effector of individual cellular pathways but also as an interface through which metabolic, regulatory, and immune signals are integrated (**Figure 3**; **Supplementary Table S1**).

The interconnected nature of ENO1 also presents a fundamental challenge for mechanistic studies. Changes in ENO1 abundance or phenotype alone cannot establish which of its activities is responsible for a given biological outcome, because alterations in catalytic activity, PTMs, localization, and molecular interactions may occur simultaneously. Separation-of-function strategies are therefore essential. When a phenotype is suspected to depend on glycolytic activity, catalytic-site mutagenesis and rescue with catalytically inactive ENO1 can distinguish metabolic from non-metabolic effects. For example, mutation of the catalytic residues K343 or K394 has been used to examine the contribution of ENO1 enzymatic activity to IAV replication (76). When PTMs are implicated, modification-site mutants provide a complementary strategy. K193Q and K193R variants, for instance, have been used to mimic or prevent ENO1 lactylation, respectively, enabling the contribution of this modification to ENO1-dependent regulation of CXCL12 expression to be examined (42).

The experimental strategy should therefore be matched to the molecular activity under investigation. Catalytic mutants, metabolic isotope tracing, and catalytically inactive rescue can help determine whether ENO1-dependent phenotypes arise from altered metabolic flux (12, 76). PTM profiling together with PTM-site mutagenesis or CRISPR-based knock-in can establish the functional significance of individual modifications under endogenous conditions (39, 42). For RNA- or DNA-associated activities, databases such as ENCORI and CatRAPID can provide candidate ENO1-associated RNAs for hypothesis generation, followed by validation using CLIP-seq or related RNA-centered approaches (42, 122, 123). ChIP-based assays can likewise be used to examine proposed DNA- or chromatin-associated functions (104). When protein interactions are implicated, Co-IP using an ENO1-specific antibody followed by mass spectrometry can identify candidate interacting proteins for subsequent validation; for example, this approach was used to identify and validate the ENO1–IFITM2 interaction during sepsis (79). Alternatively, bimolecular fluorescence complementation (BiFC) can assess protein–protein interactions in living cells, as demonstrated for the interaction between ENO1 and the Cryptosporidium parvum sporozoite surface protein Cpgp40 (12). Compartment-specific manipulation, advanced imaging, proximity labeling, and interactome analysis can further define the spatial and dynamic organization of ENO1-associated networks (12, 49). Integrating these approaches with genetic rescue and separation-of-function experiments will help distinguish primary ENO1 activities from secondary consequences and establish causal links between specific molecular states and infection-associated phenotypes. Besides, temporal remodeling of ENO1 during infection remains poorly characterized. Future studies integrating time-resolved analyses of ENO1 post-translational modifications, subcellular localization, and molecular interactions may determine whether its functional states are dynamically reshaped across different stages of infection. Importantly, ENO1 functions may also vary substantially across different cell types and tissue contexts during infection, rather than following a universal functional pattern. Systematic characterization of cell-type-specific ENO1 functions will therefore be important for understanding how its functional states contribute to host defense and pathogen infection.

The functional plasticity of enolase extends beyond host ENO1 to pathogen-derived enolases. Across diverse microorganisms, enolases retain their conserved glycolytic role while contributing to microbial adaptation, adhesion, tissue invasion, plasminogen recruitment, immune modulation, and pathogen-specific regulation (**Supplementary Table S1**). Host ENO1 and pathogen-derived enolases therefore share a conserved molecular framework but have diversified according to their respective cellular environments, interaction networks, and evolutionary pressures (**Table 2**, **Figure 1**). Their comparison highlights an important feature of the enolase family: conservation of a metabolic and structural core can coexist with substantial functional diversification. Evolutionary conservation also creates an important intersection between enolase biology and disease. Pathogen-derived enolases share structural similarities with host ENO1 and can elicit immune responses. In some cases, this may lead to immune cross-reactivity and molecular mimicry, potentially contributing to infection-associated autoimmunity (24, 112, 124, 125). At the same time, their extracellular and host-interacting activities may provide opportunities for therapeutic intervention. Enolase-based vaccination has shown protective effects in experimental models of *Candida albicans* and *Leishmania donovani* infection (107–109), while disruption of the *Candida albicans* Eno1–plasminogen interaction reduces fungal invasion and enhances antifungal efficacy (22). Conversely, inhibitors targeting *Mycobacterium tuberculosis* enolase may also suppress human α-enolase, illustrating the challenge of targeting conserved catalytic sites (94). These findings support strategies directed toward pathogen-specific structural features, extracellular domains, or virulence-associated molecular interactions rather than broadly targeting conserved enolase activity.

**Table 2:** Comparison of host ENO1 and pathogen-derived enolases.

Functional feature	Host ENO1	Bacterial enolases	Fungal enolases	Protozoan/parasitic enolases
Localization	Cytoplasm; also nucleus, plasma membrane, mitochondria, pathogen-containing compartments, and extracellular space	Cytoplasm; frequently also surface-associated or extracellular	Cytoplasm; cell wall, surface, and extracellular space	Mainly cytoplasm; may also localize to parasite surface or extracellular compartments.
Interacting partners	Host proteins, DNA/RNA, signaling molecules, and proteins of pathogens	Host or bacterial proteins involved in adhesion, invasion, RNA regulation, and metabolism	Host proteins involved in plasminogen binding, complement regulation, and immune activation	Host receptors, plasminogen, and parasite proteins involved in invasion and host interaction.
Metabolic functions	Glycolysis and regulation of host metabolic flux	Glycolysis and pathogen metabolic fitness	Glycolysis, growth, and viability	Glycolysis, energy metabolism, and parasite development.
Immune effects	Regulates innate immune signaling, inflammation, and immune-cell functions	Immune evasion and modulation of host inflammatory responses	Modulates complement, antibody responses, and inflammation	Can influence host immune responses and may contribute to immune evasion.
Virulence mechanisms	Not a pathogen-encoded virulence factor; can be exploited by pathogens to support replication or intracellular survival	Adhesion, invasion, plasminogen recruitment, immune evasion, and persistence	Adhesion/invasion, plasminogen recruitment, complement evasion, and dissemination	Invasion, tissue attachment, plasminogen recruitment, and host interaction.
Therapeutic implications	Host targeting may affect essential metabolic functions and cause toxicity	Potential pathogen-directed targets, but similarity to host ENO1 may cause off-target effects	Potential targets or vaccine candidates; cross-reactivity with host ENO1 requires consideration	Potential vaccine or therapeutic targets; molecular conservation may limit selectivity.

For host ENO1, however, the therapeutic challenge is different. Because ENO1 performs an essential role in glycolysis, a universal therapeutic window for its inhibition cannot currently be defined. The metabolic consequences of ENO1 suppression are likely to depend on the extent and duration of inhibition, as well as cellular and tissue metabolic demands. Therapeutic strategies may therefore need to preserve sufficient basal glycolytic activity while selectively interfering with infection-associated non-metabolic activities. Partial or transient inhibition, compartment-selective intervention, and disruption of disease-associated PTMs or molecular interactions represent potential approaches to achieving this balance. The central therapeutic challenge is consequently not simply to inhibit ENO1, but to determine which ENO1-dependent activity should be targeted, in which cellular compartment, and at what stage of infection (**Table 1**).

Future research should move beyond asking whether ENO1 is involved in infection toward determining how its distinct molecular states are established, interconnected, and translated into specific biological outcomes. A central objective will be to define the principles governing the transition of ENO1 between metabolic and non-metabolic activities and to understand how these states are shaped by cellular stimuli, metabolic conditions, PTMs, subcellular localization, and molecular interactions. Such an integrated view may reveal disease-associated ENO1 states that can be selectively manipulated without disrupting its essential metabolic role. In parallel, comparative investigation of host and pathogen-derived enolases may distinguish conserved mechanisms from pathogen-specific vulnerabilities and providing a basis for selective intervention at the host–pathogen interface. Ultimately, understanding ENO1 as an interconnected regulatory hub may reveal opportunities to selectively manipulate infection-associated functions while preserving its essential metabolic role.
